# Supplementary material for: A novel WD40-repeat protein involved in formation of epidermal bladder cells in the halophyte quinoa
Source: Commun Biol. 2020 Sep 17;3:513. doi: 10.1038/s42003-020-01249-w (PMC7498606; doi:10.1038/s42003-020-01249-w)
Supplement: Supplementary file 1 — Supplementary Information [file 42003_2020_1249_MOESM1_ESM.pdf]

Supplementary figures

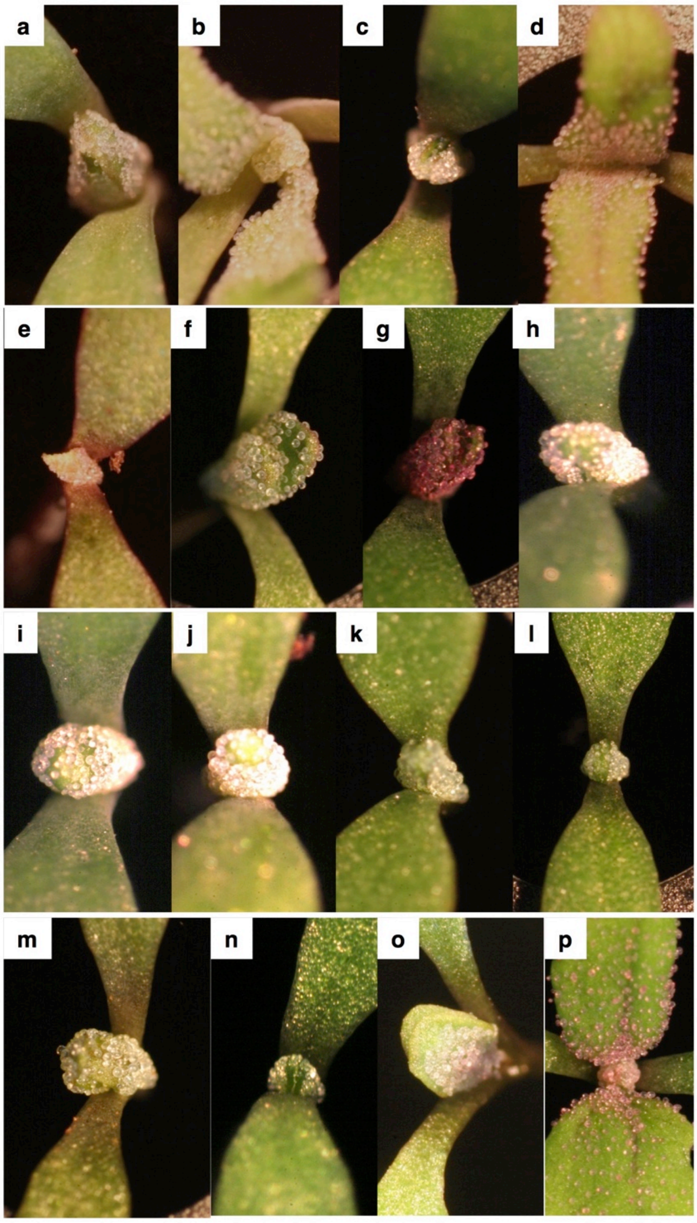

Supplementary Fig. 1: Shoot apices of *Chenopodium* species One-week-old seedlings of *Chenopodium berlandieri* subsp. *nuttalliae* (**a**–**c**), *C. formosanum* (**d**, **e**), *C. giganteum* (**f**, **g**), *C. pallidicaule* (**h**–**j**), *C. leptophyllum* (**k**), *C. neomexicanum* (**l**), *C. fremontii* var. *pringlei* (**m**), *C. strictum* (**n**), *C. glaucum* (**o**), and *C. album* L. var. *centrorubrum* Makino (**p**). Accessions numbers for these *Chenopodium* species are listed in Supplementary Table 7.

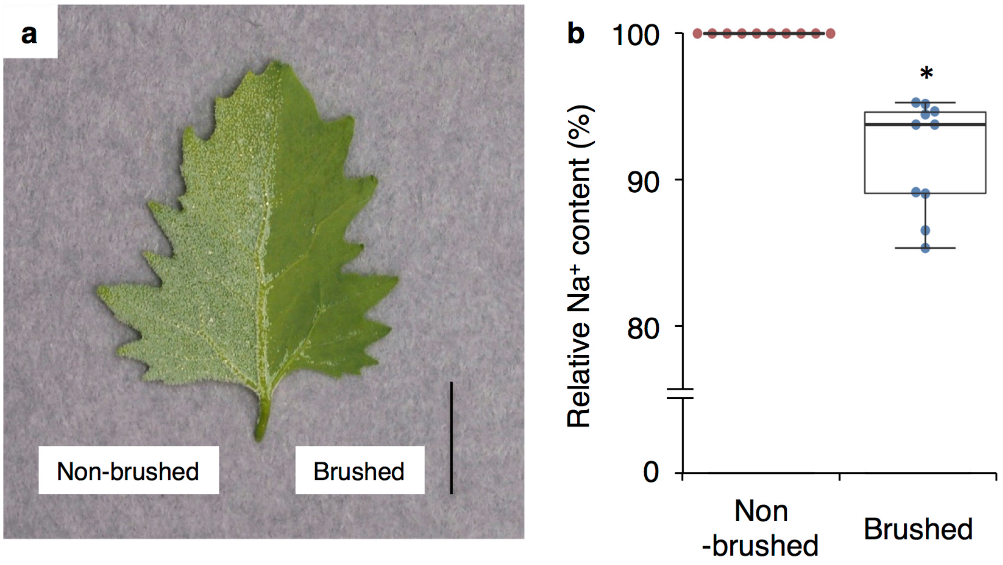

Supplementary Fig. 2: Salt stress treatment of quinoa Brushed leaf of salt-treated wild-type (WT) plant (**a**). "Brushed" indicates removal of EBCs from the leaf. Bar = 1 cm. Relative Na^+ content of leaf from salt-treated WT plants (**b**). Error bar represents mean ± SD (n = 10). *$p < 0.05$ vs. Non-brushed leaf. Data points are available in Supplementary Data 5.

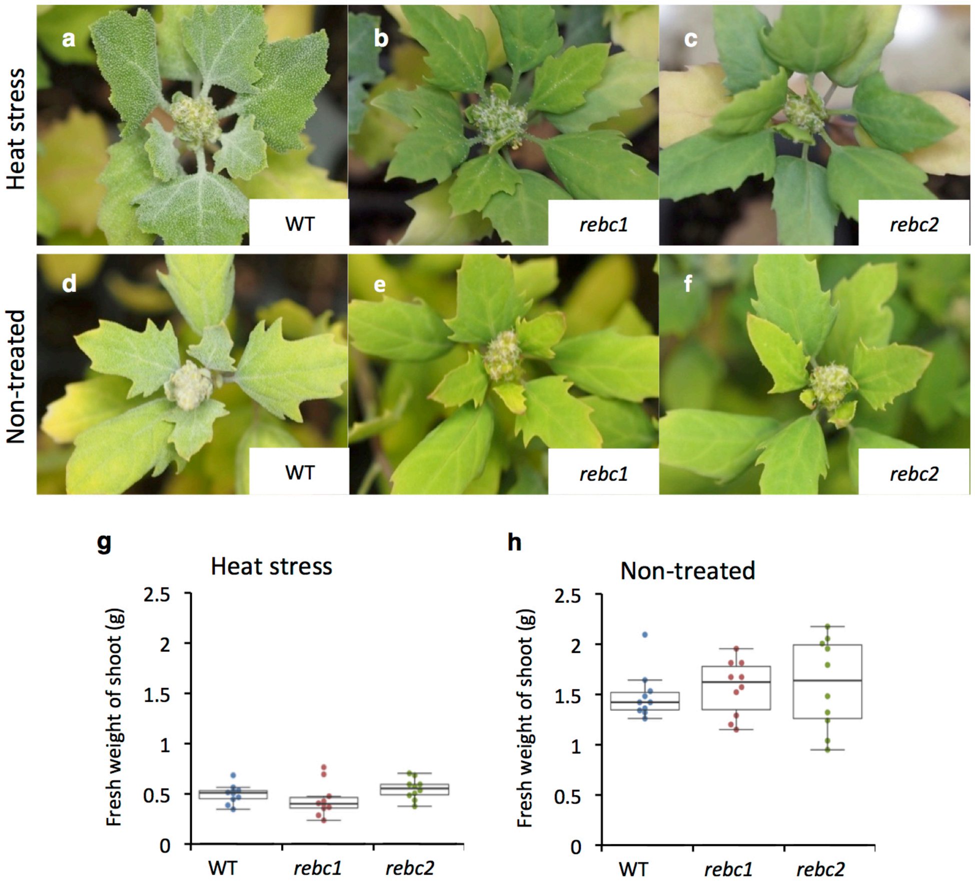

Supplementary Fig. 3: High-temperature treatment of quinoa Wild-type (WT; **a**), *rebc1* (**b**), and *rebc2* (**c**) plants after 2 weeks of heat treatment. Non-treated WT (**d**), *rebc1* (**e**), and *rebc2* (**f**) plants. Fresh weights of shoots of heat-treated (**g**) and non-treated plants (**h**). Error bars represent means ± SD. Data points are available in Supplementary Data 5.

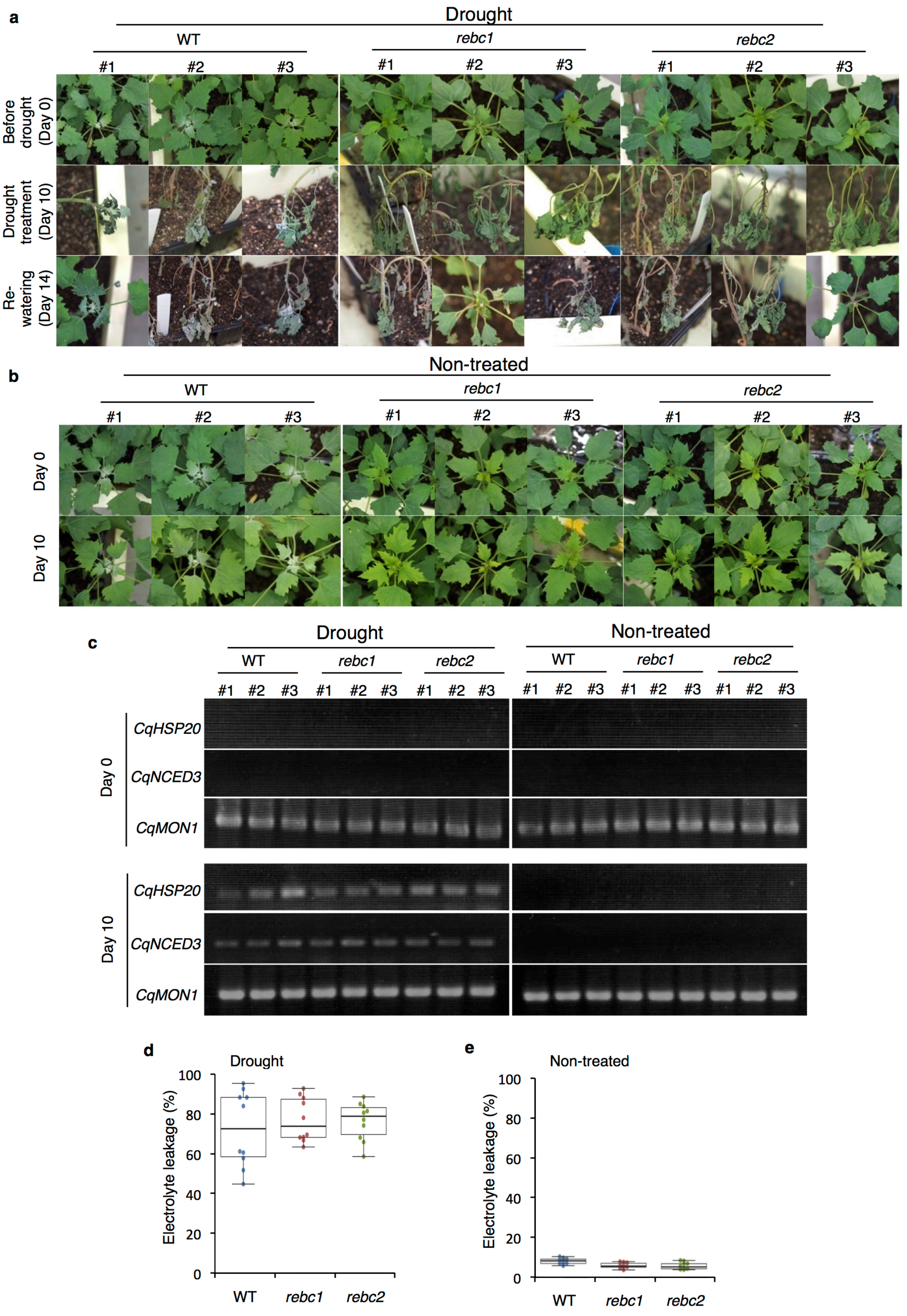

Supplementary Fig. 4: Drought-stress treatment of quinoa Photograph of quinoa plants before drought treatment (upper panels), after 10 days of drought (middle panels), and after 4 days of recovery (lower panels)

(**a**). Photograph of non-treated plants on day 0 (upper panels) and day 10 (lower panels) (**b**). Expression analysis of drought stress–induced genes (**c**). *CqHSP20* and *CqNCED3* are drought-stress induced genes in quinoa. *CqMON1* is an internal control. #1, #2, and #3 indicate individual index numbers of plants. Electrolyte leakage induced by drought (**d**) or non-treated (**e**) in leaf tissues of quinoa plants. Error bars represent means ± SD (n = 10). Data points are available in Supplementary Data 5.

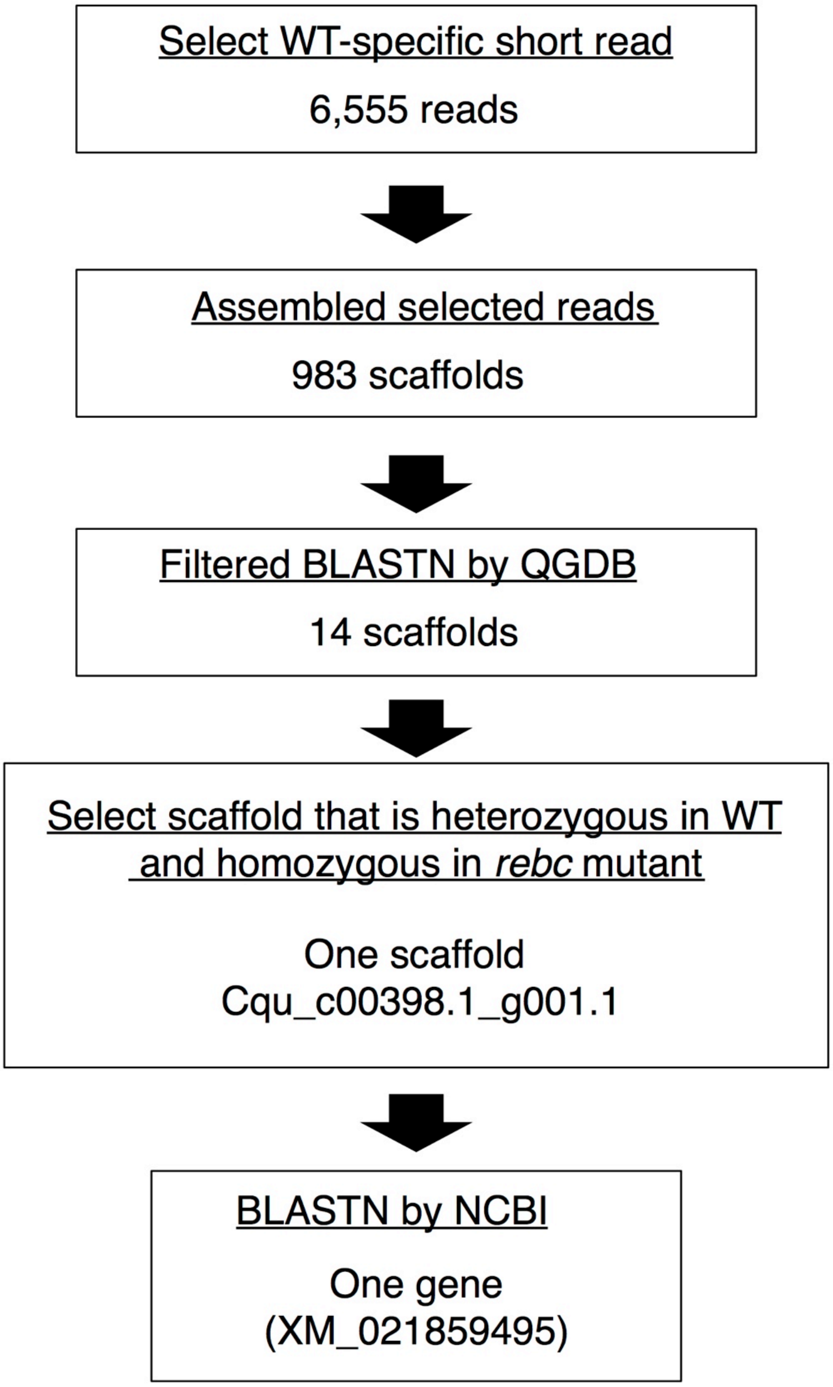

Supplementary Fig. 5: Flow chart for identifying genes using the *in silico* subtraction method

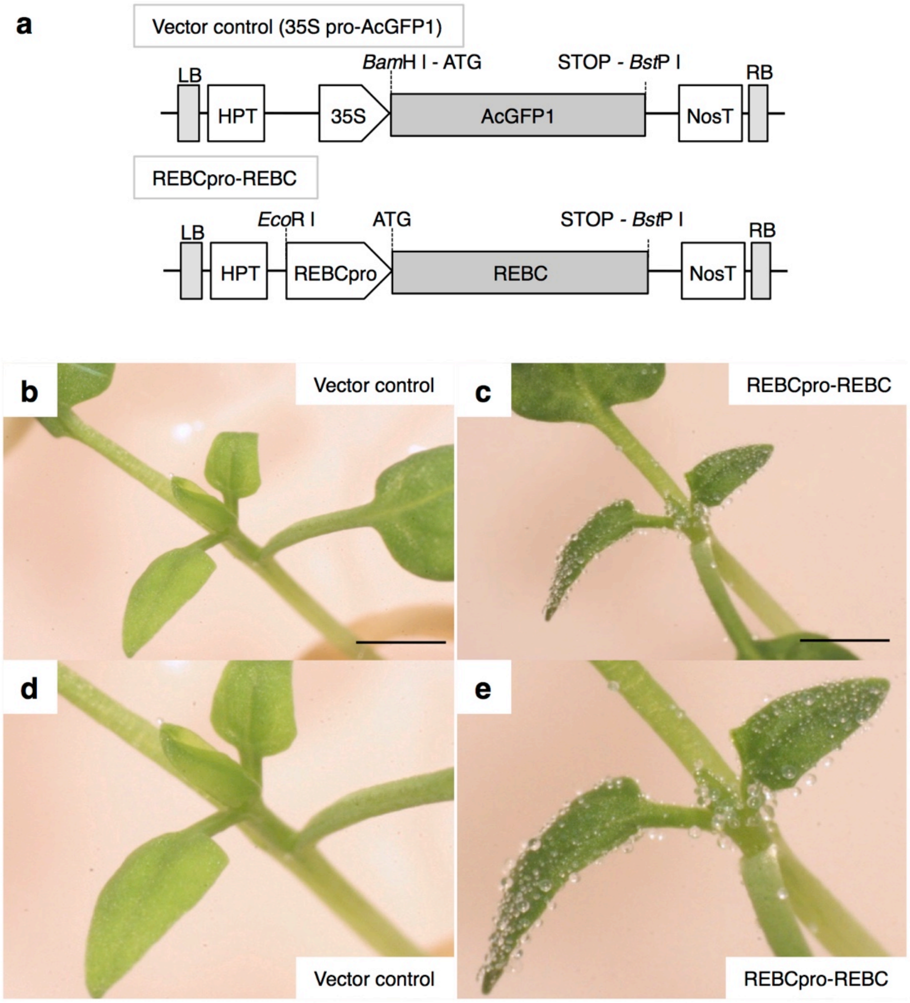

Supplementary Fig. 6: Complementation test using *Rhizobium rhizogenes* Schematic representations of plant expression vectors (**a**). REBC, *REBC* coding sequence (CDS); AcGFP1, *AcGFP1* CDS; 35S, CaMV 35S promoter; REBC pro, *REBC* promoter; NosT, *nopaline synthase* terminator; RB, right border; LB, left border; HPT, *hygromycin phosphotransferase* expression cassette; ATG, start codon; STOP, stop codon. Vector control line (**b**, **d**) and *REBC* expression line (**c**, **e**). (**d**) and (**e**) show close-up views of (**b**) and (**c**), respectively. Bars = 4 mm.

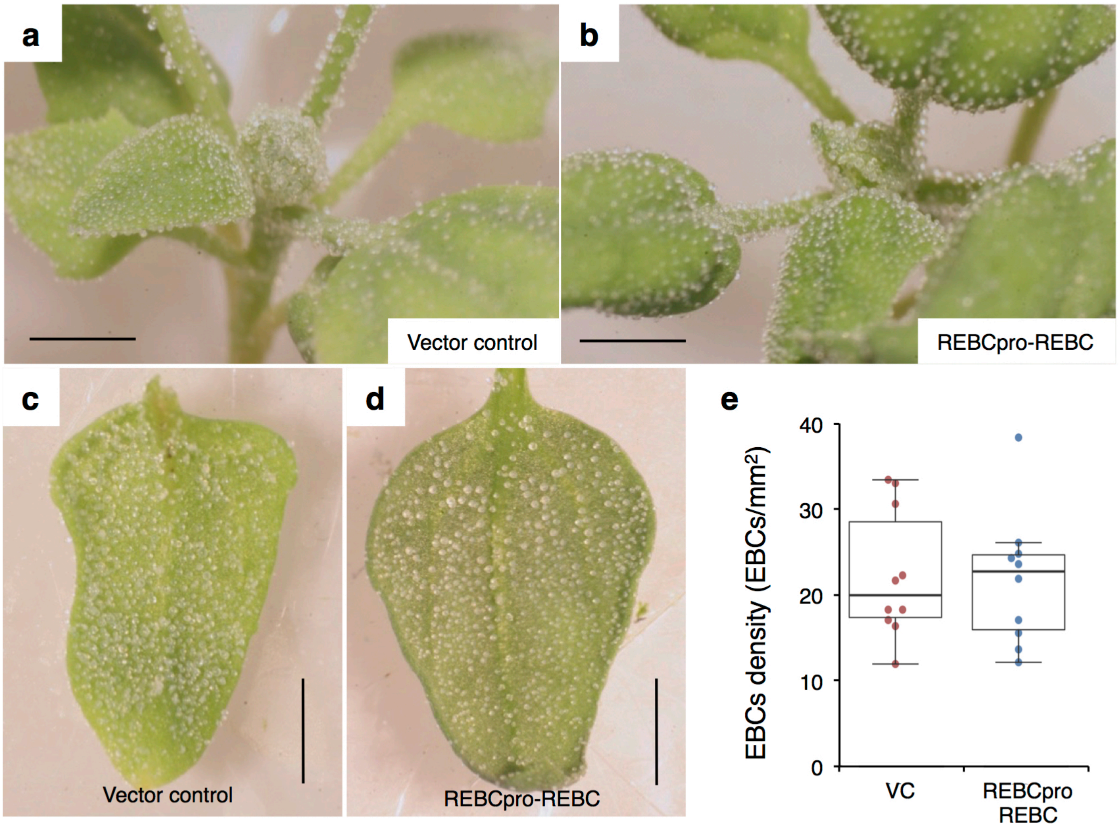

Supplementary Fig. 7: Transient expression using *Rhizobium rhizogenes* Transient expression line of vector control (VC) (**a**, **c**) and REBCpro-REBC (**b**, **d**) in wild-type (WT) quinoa. (**a, b**) and (**c, d**) show an infected plant and leaf, respectively. Bars = 2 mm. EBC density of infected leaves in the WT (**e**). Error bars represent means ± SD (n = 10). Data points are available in Supplementary Data 5.

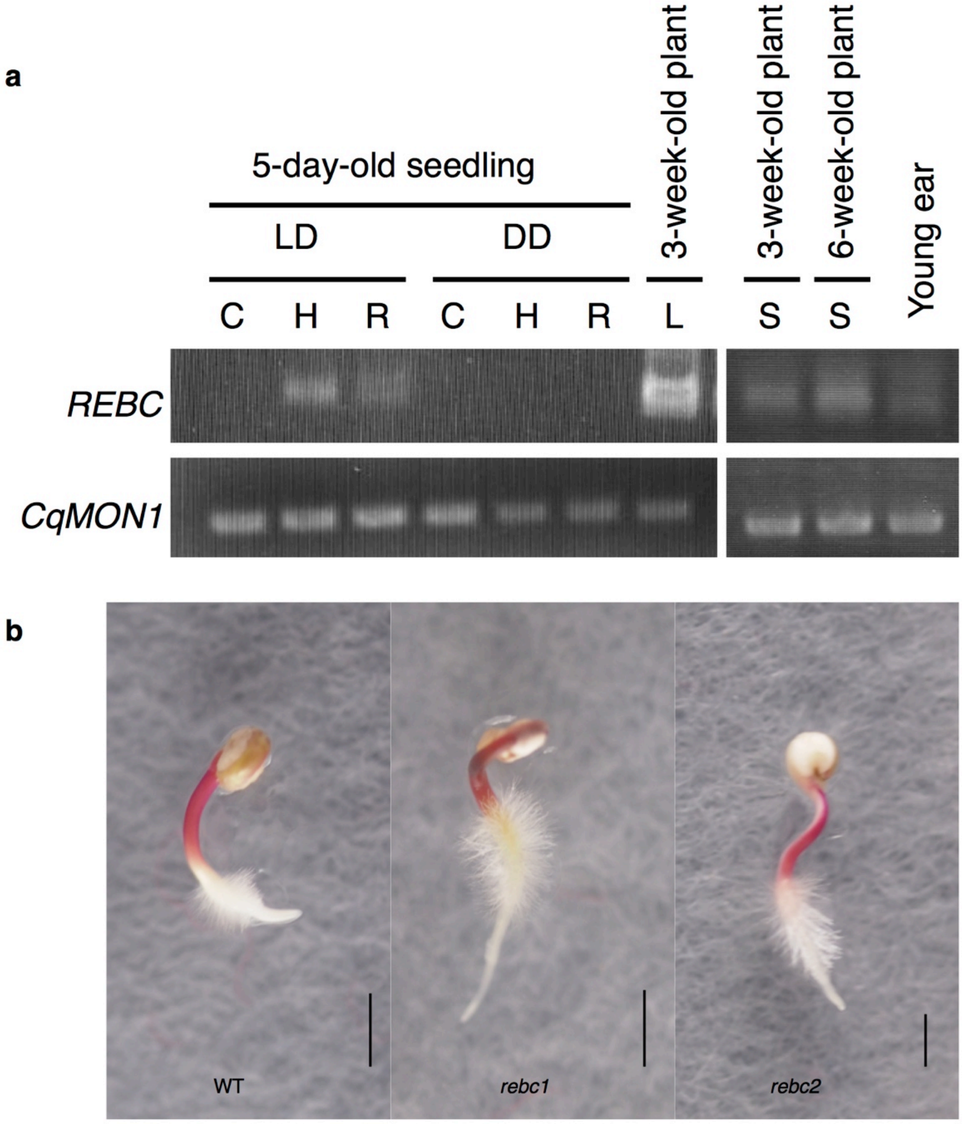

Supplementary Fig. 8: Analysis of *REBC* expression in quinoa plants RT-PCR analysis of *REBC* expression (**a**). **“**LD” and “DD” indicate a light/dark cycle (light/dark = 12-h / 12-h) and continuous dark conditions, respectively. “C”, “H”, “R”, “S”, and “L” indicate expression in the cotyledons, hypocotyls, roots, shoot apices, and leaves, respectively. *CqMON1* is an internal control. (**b**) Photograph of 5-day-old seedlings. WT indicates wild-type. Bars = 2 mm.

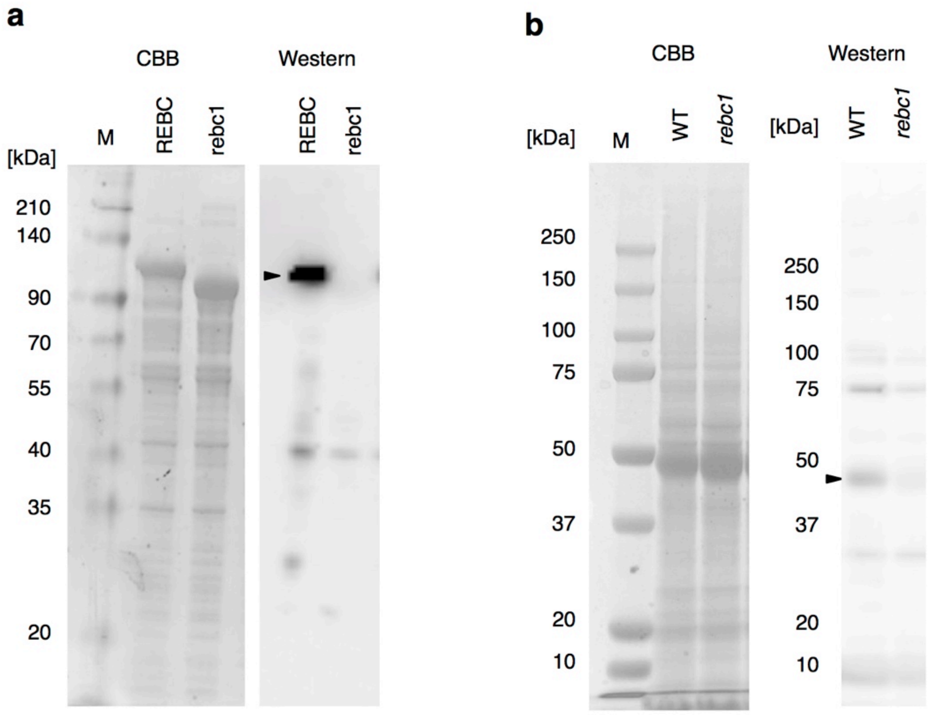

Supplementary Fig. 9: Detection of REBC protein by western blotting (**a**) Detection of REBC fusion protein from *E. coli* extracts. REBC, trigger factor (TF)-REBC fusion protein; rebc1, TF-rebc1 fusion protein. (**b**) Detection of REBC protein from plant extracts. WT, wild-type plant extract; *rebc1*, *rebc1* mutant extract. Right panel, Coomassie brilliant blue (CBB) staining; left panel, western blotting (Western). Arrowheads indicate REBC fusion or REBC protein. M, protein size marker. Numbers indicate molecular weights (kDa).

```
REBC      MHAHTKISLVHISTPVFSLAMDRQLSHKKSLSCFFDEDFTEDQSPSFQHLSKHNQNFLAR 60
AtTTG1    ----------MDNSAPDSLSRSETAVTYDSPYPLYAMAFSSLRSSSGHRIAVG--SFLED 48
                      .:.  **: ..     .*   ::   *:. :*.* ::::    .**

REBC      LHGTHFYPDTSPMMSQSPESSWSPSPSLTPSHPSLLYCCISSLRRDGDIYSLTVFGDLVL 120
AtTTG1    YN-----NRIDILSFDSDSMTVKPLPNLSFEHP--------------------------- 76
           :        . :  :* . : .* *.*: .**

REBC      TGSSSRRVYAWQSLDCHAKGYIQSSSGEVRAMQVYDDMLFTAHKDHKIRIWNMRTCSGSF 180
AtTTG1    -YPPTKLMFSPPSLRRPSSGDLLASSG------------------DFLRLWEINEDSSTV 117
            ..:: :::  **   :.* : :***                  . :*:*::.  *.:.

REBC      RARKVLTLPCASHFKSFICRSVVPHHRLTANKPITPQHRDIISCMAFYYVESILYTGSFD 240
AtTTG1    EPISVLNNSKTSEFCAPLTS--------FDWNDVEPKR---------------LGTCSID 154
          .. .**. . :*.* : :             : : *::               * * *:*

REBC      KTIKAWKLSVKKCIDSFVAHGDHINDMVVNQQSGYLFTCSSDGTVKMWLRVYGEHSHVLI 300
AtTTG1    TTCTIWDIEKSVVETQLIAHDKEVHDIAWG-EARVFASVSADGSVRIFDLRDKEHSTIIY 213
          .* . *.:. .    .::**...::*:. . ::  : : *:**:*:::     *** ::

REBC      KVFSFHTYPIYALALGVSPSQRSFLYSGSSDGCINFWVQEISTHYNHGGVLEGHQFAVLC 360
AtTTG1    ESPQPDT-PLLRLAWNKQDLRYMATILMDSNKVVILDIRSPTMPVAELERHQASVNAIAW 272
          :  . .* *:  ** . .  :       .*:  : : ::. :    .    :.   *:

REBC      LVTLDNLVISGSEDSTIRIWRREKVRFTHECLAVLEGHRGPVRCLAASLQDELVTSFLVY 420
AtTTG1    APQSCKHICSGGDDTQALIWE----------LPTVAGPNG-------------IDPMSVY 309
               : : **.:*:   **.          *..: * .*             : .: **

REBC      SASLDQTFKVWRVKLLREMKKSPGRHSNGDDDTEDTNSAGCEPSPVLSPSWVKKKLQCRS 480
AtTTG1    SAGSEINQLQWSS----------------------------SQPDWIGIAFANKMQLLRV 341
          **. : .   *                              . .  :. ::.:*    *

REBC      LK 482
AtTTG1    --
```

Supplementary Fig. 10: Alignment of the deduced amino acid sequences of REBC and *Arabidopsis* TTG1 (AtTTG1)

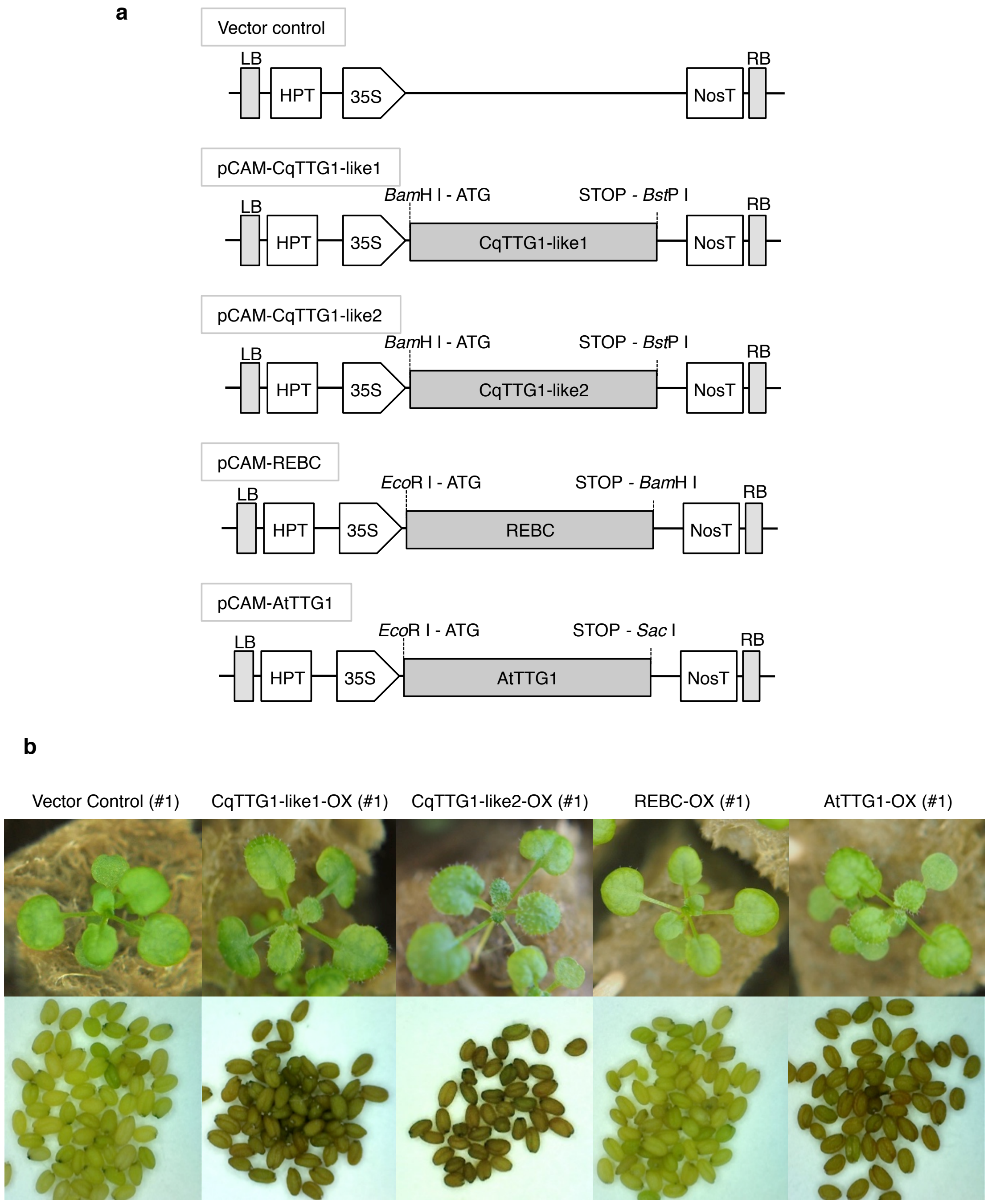

Supplementary Fig. 11: Complementation test using *Arabidopsis ttg1-21* mutant (**a**) Schematic representations of plant expression vectors. CqTTG1-like1, *CqTTG1-like1* coding sequence (CDS); CqTTG1-like2, *CqTTG1-like2* CDS; REBC, *REBC* CDS; AtTTG1, *AtTTG1* CDS; 35S, CaMV 35S promoter; NosT, *nopaline synthase* terminator; RB, right border; LB, left border; HPT, *hygromycin phosphotransferase* expression cassette; ATG, start codon; STOP, stop codon. (**b**) Complementation test

using an *Arabidopsis ttg1-21* mutant. Transgenic plants (upper panel) and seeds (lower panel) for vector control, *CqTTG1-like1* overexpression line (CqTTG1-like1-OX), *CqTTG1-like2* overexpression line (CqTTG1-like2-OX), *REBC* overexpression line (REBC-OX), and *AtTTG1* overexpression line (AtTTG1-OX) are shown. #1 is the individual index number of these transgenic lines.

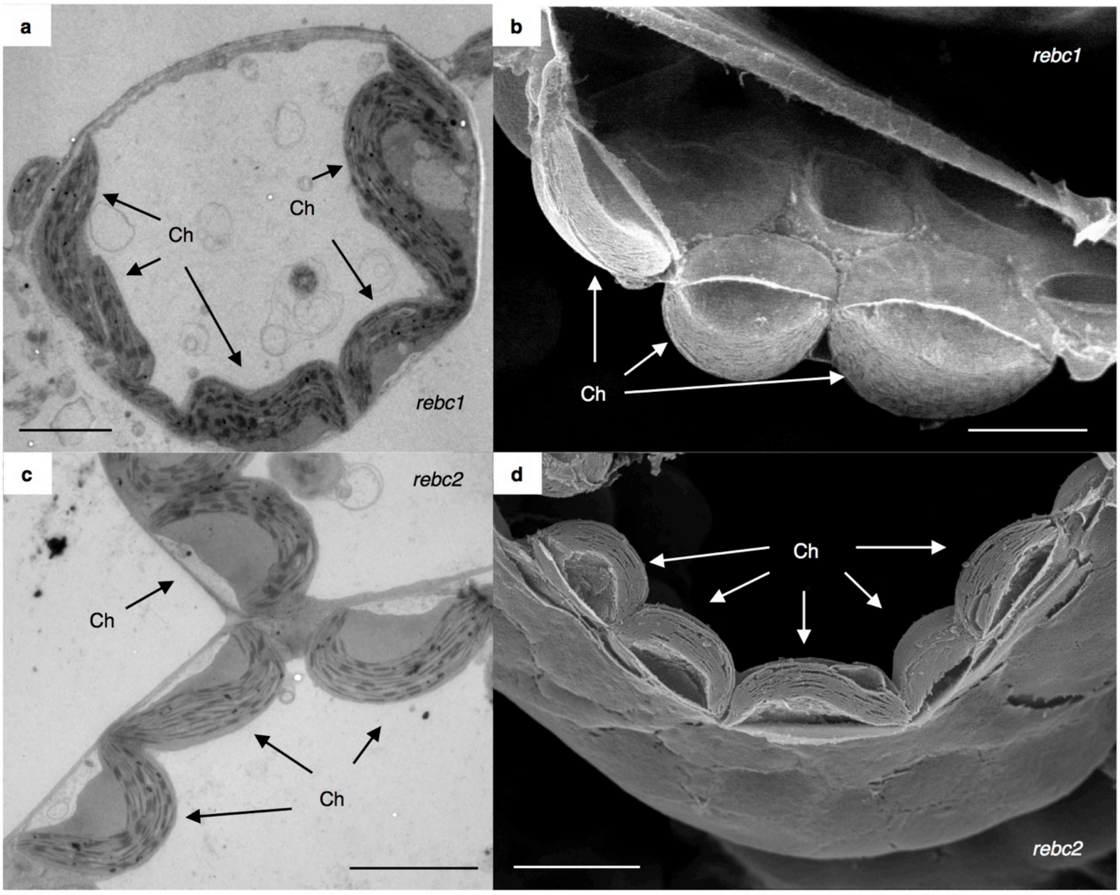

Supplementary Fig. 12: Morphology of chloroplasts in quinoa Transmission electron microscopy (**a** and **c**) and scanning electron microscopy (**b** and **d**) images of chloroplasts in *rebc* mutants. (**a**) and (**b**) show chloroplasts of *rebc1* mutants. (**c**) and (**d**) show chloroplasts of *rebc2* mutants. Ch, chloroplast. Bars = 5 µm.

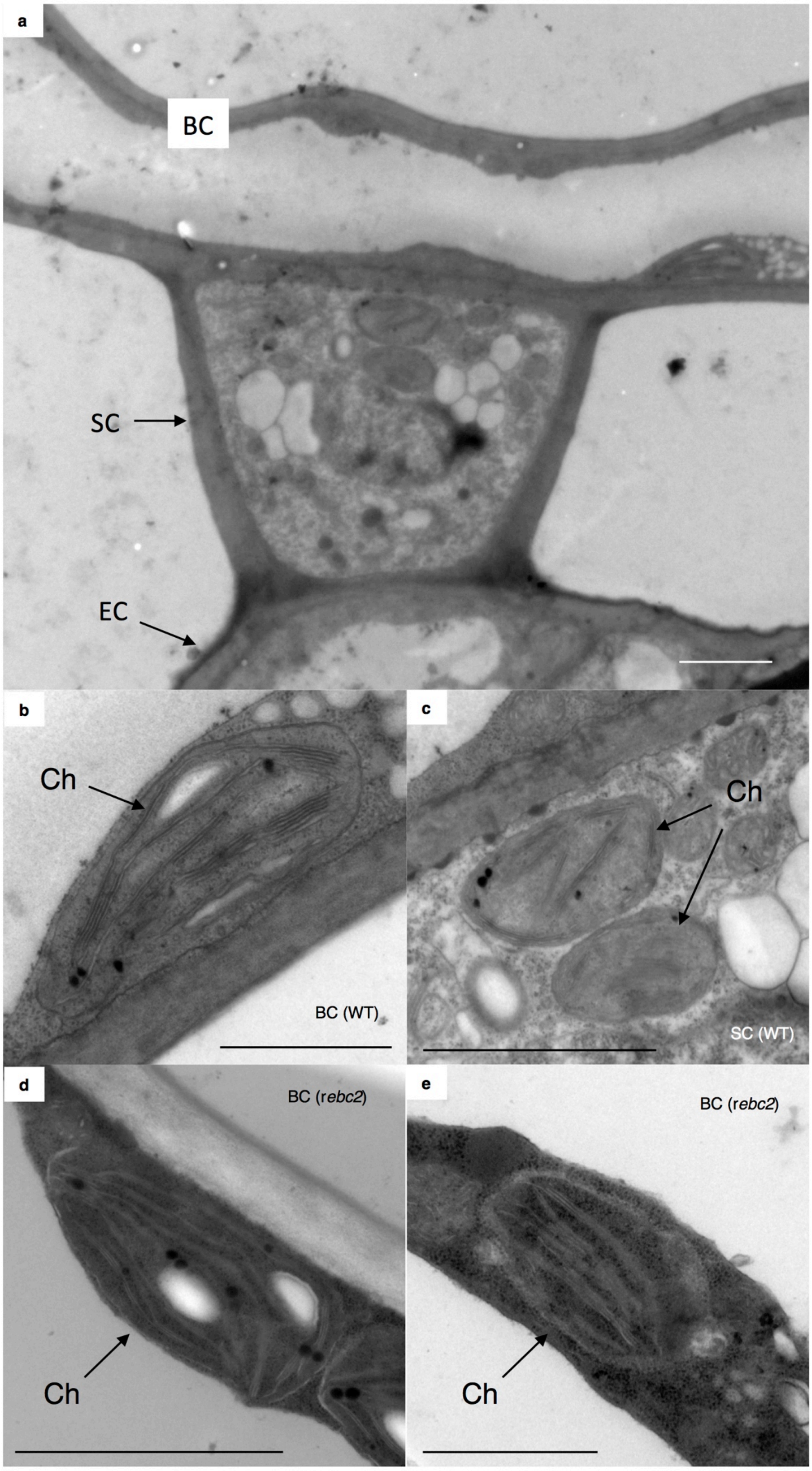

Supplementary Fig. 13: TEM image of chloroplast morphology in quinoa Transmission electron microscopy image of an EBC (**a**), chloroplasts in a bladder cell (BC) (**b, d, e**), and chloroplasts in a stalk cell

(SC) (**c**). **b** and **c** show chloroplasts of WT. **d** and **e** show chloroplasts of a *rebc2* mutant. EC, epidermal cell; Ch, chloroplast. Bars = 2 µm (**a**–**d**) and 1 µm (**e**).

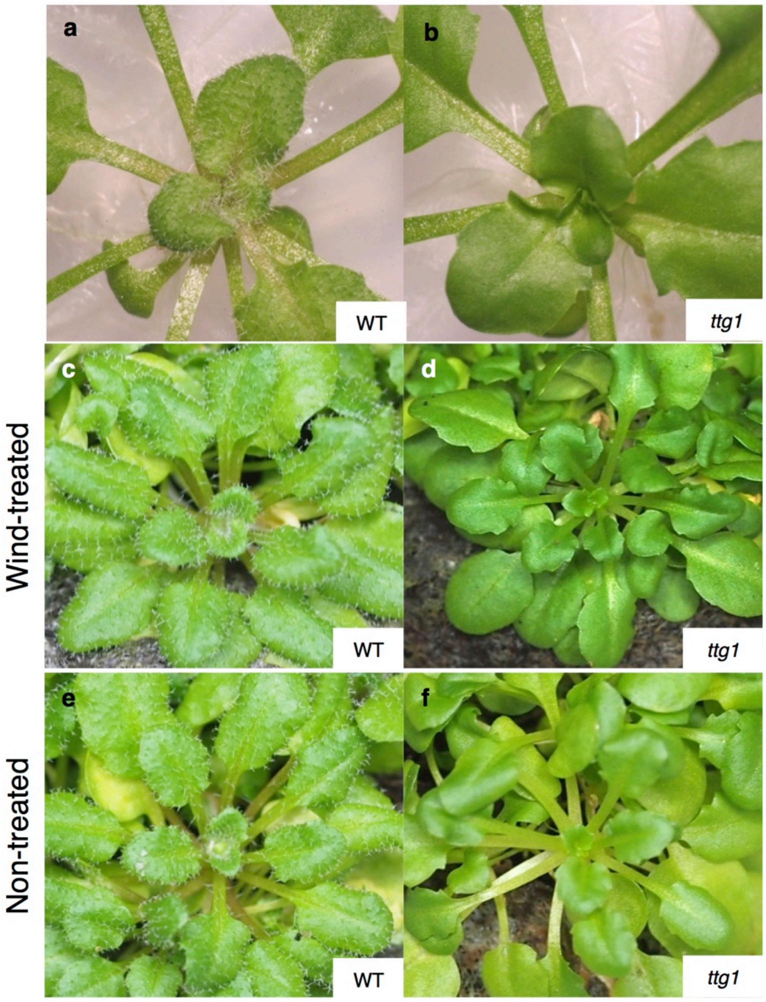

g

		Total plants	Damaged shoot apex
Wind stress	WT	10	0
	ttg1	10	0

Supplementary Fig. 14: Abiotic-stress treatment of *Arabidopsis* Four-week-old *Arabidopsis* wild-type (WT, **a**) and *ttg1-21* mutant (**b**) plants. WT (**c**) and *ttg1* (**d**) plants after wind treatment. Non-treated WT (**e**) and *ttg1* (**f**) plants. Summary of effects of wind stress treatments (**g**).

Supplementary Tables

Supplementary Table 1

List of the number of EBC in quinoa leaves

	Number of EBC per leaf (mean ± SD)
WT	1158.6 ± 176.9
rebc1 mutant	2.9 ± 2.1
rebc2 mutant	3.7 ± 3.0
F1 generation of *rebc1* (homo) ×*rebc2* (homo)	2.7 ± 2.5

Data are available in Supplementary Data 5.

Supplementary Table 2

Summary of effect of abiotic-stress treatment

Abiotic treatment	Plant	Total plants	Damaged shoot apex*	Partially damaged shoot apex*
Wind	WT	18	0	0
	rebc1	18	18	0
	rebc2	16	16	0
UV-B	WT	14	0	8
	rebc1	13	10	3
	rebc2	14	10	4
High-salinity	WT	15	0	0
	rebc1	15	0	0
	rebc2	15	0	0
High-temperature	WT	14	0	0
	rebc1	14	0	0
	rebc2	14	0	0

*: "Damaged" and "partially damaged" indicate entirely and partially damaged shoot apices, respectively

Supplementary Table 3

Summary of drought-stress treatment

Plant	Total plants	Suvived plants
WT	15	4
rebc1	15	4
rebc2	15	2

Supplementary Table 4

Summary of segregation test

	Total	*rebc* (Mutant type)	WT (Wild type)	Hypothesis (*rebe* : WT)	χ2 *	p-value
M_3 generation of *rsbc1* mutant	98	22	76	1 : 3	0.304	0.559
M_3 generation of *rebc2* mutant	144	37	107	1 : 3	0.370	0.847
F1 *generation of* *rebc1* (homo) ×*rebc2* (homo)	8	8	0	-	-	-

* Chi-square values with a significance level of 0.05

Supplementary Table 5

Summary of paired-end sequence reads obtained from Illumina sequencing

Sample	Number of paired-end reads	Read length (bp)	Total sequence (Mb)	Illumina Sequencer used	GenBank Accession number
WT bulk of *rebc1* heterozygous line	1,696,213[a]	250	121.6	MiSeq	DRX163715
rebc bulk of *rebc1* heterozygous line	1,882,340[a]	250	133.5	MiSeq	DRX163716
WT bulk of *rebc2* heterozygous line	2,152,825[a]	250	149.3	MiSeq	DRX163717
rebc bulk of *rebc2* heterozygous line	2,305,649[a]	250	149.4	MiSeq	DRX163718
rebc1 mutant bulk	252,426,628	302	76,232.8	HiseqX	DRX138189
WT bulk	207,044,841	302	62,527.5	HiseqX	DRX138190

[a]Sequence reads having more than 20% of their sequenced nucleotides with phred quality score of less than 20 were excluded.

Supplementary Table 6

Summary of mapping by sequencing results

QGDB contig *1	Position	Ref base	SNP	SNP index	Depth		Gene annotation	Phytozome12 accession *2
Contig24_consensus	103581	C	Y	0.64	37	intron	Indole-3-glycerol phosphate synthase like	AUR62039696
Contig12941_consensus	42624	C	Y	0.72	37	intergenic		
Contig21533_consensus	5169	T	Y	0.67	34	intergenic		
Contig3444_consensus	109079	A	R	0.61	34	intergenic		
Contig95_consensus	118998	T	Y	0.6	30	intergenic		
Contig9064_consensus	14913	A	R	0.73	30	intergenic		
Contig3820_consensus	71450	A	R	0.73	30	intergenic		
Contig4254_consensus	32426	A	R	0.64	28	intergenic		
Contig4313_consensus	114681	C	Y	0.62	27	intron	Probable E3 ubiquitin ligase SUD1	AUR62023210
Contig2262_consensus	96216	A	R	0.7	27	intergenic		
Contig11586_consensus	23195	C	Y	0.61	26	intergenic		
Contig6325_consensus	54702	A	R	0.76	26	intergenic		
Contig4721_consensus	21924	A	R	0.65	26	intergenic		
Contig12838_consensus	24339	C	Y	0.8	25	intergenic		
Contig6166_consensus	27297	T	Y	0.76	25	intergenic		
Contig2262_consensus	19972	C	Y	0.6	25	intergenic		
Contig2262_consensus	127035	C	Y	0.72	25	intron	Amino acid permease 8 like	AUR62040229
Contig8203_consensus	13013	A	R	0.72	25	intron	Thioredoxin-related transmembrane protein 2 like	AUR62042442
Contig1648_consensus	42792	A	R	0.75	24	intergenic		
Contig1407_consensus	51046	C	Y	0.62	24	intergenic		
Contig4356_consensus	20847	G	R	0.7	24	intron	Protein OSB1like	AUR62032259
Contig5175_consensus	14919	A	R	0.62	24	intergenic		
Contig7433_consensus	12464	G	R	0.73	23	intergenic		
Contig2262_consensus	85231	C	Y	0.6	23	intergenic		
Contig9197_consensus	6924	G	R	0.6	23	intergenic		
Contig24_consensus	222730	A	R	0.63	22	intergenic		
Contig2487_consensus	110630	T	W	0.63	22	intergenic		
Contig9469_consensus	19868	T	Y	0.71	21	intron	Protein of unknown function	AUR62023643
Contig7439_consensus	31218	T	C	0.95	21	intergenic		
Contig3789_consensus	30169	G	R	0.6	20	intergenic		
Contig14105_consensus	39138	A	R	0.65	20	intergenic		
Contig3378_consensus	55649	C	Y	0.6	20	intergenic		
Contig11824_consensus	35814	T	Y	0.6	20	intergenic		
Contig7439_consensus	31234	C	T	0.95	20	intergenic		
Contig4492_consensus	52447	G	K	0.75	20	intergenic		

*1 : QGDB quinnoa genome data base (http://quinoa.kazusa.or.jp/)

*2 : Pytozome12 (https://phytozome.jgi.doe.gov/pz/portal.html#!info?alias=Org_Cquinoa_er)

Supplementary Table 7

Chenopodium spp. used in this study

Label in Supplemental Fig. 1	Taxonomy	Country of origin	Plant name	Accession	
-	*C. quinoa*	Bolivia, La Paz	CQ127	PI 614927	United States Department of Agriculture (USDA)
A	*C. berlandieri* subsp. *nuttalliae*	Mexico, Jalisco	-	PI 433229	USDA
B	*C. berlandieri* subsp. *nuttalliae*	Mexico	Santa Elena 7	PI 476820	USDA
C	*C. berlandieri* subsp. *nuttalliae*	Mexico	huautzontle	PI 568155	USDA
D	*C. formosanum*	Taiwan	Balt	PI 433378	USDA
E	*C. formosanum*	Taiwan	Balt	PI 433379	USDA
F	*C. giganteum*	India, Arunachal Pradesh	Balamun	PI 667180	USDA
G	*C. giganteum*	Netherlands	PLANT VIRUS	PI 677102	USDA
H	*C. pallidicaule*	Peru, Puno	Chupica Canihua (Aymara), Canihua Rojo	PI 510525	USDA
I	*C. pallidicaule*	Peru	Chupica Canihua (Aymara), Canihua Blanco	PI 510526	USDA
J	*C. pallidicaule*	Bolivia, La Paz	Line 0142	PI 665281	USDA
K	*C. leptophyllum*	United States, Nevada	BYU834	Ames 29780	USDA
L	*C. neomexicanum*	United States, Utah	BYU527	PI 666317	USDA
M	*C. fremontii* var. *pringlei*	United States, Utah	BYU202	Ames 27373	USDA
N	*C. strictum*	United States, Utah	BYU598	PI 666324	USDA
O	*C. glaucum*	-	-	9548S	Institute of Plant Science and Resources, Okayama University
P	*C. album* L. var. *centrorubrum* Makino	-	-	4546S	Institute of Plant Science and Resources, Okayama University

Supplementary Table 8
Primers used in this study

			Sequence (5´→ 3´)
Vector construction			
Overexpression	*REBC*	Forward	AAAAGATCTATGCACGCACACACAAAGATTTCC
		Reverse	AAAGGTCACCCTATTTAAGACTACGACATTGAAG
	CqTTG1-like1	Forward	AAAGGATCCATGGAGAATTCAACCCAAGAATCTCACCTC
		Reverse	AAAGGTCACCTCAAACTCTCAGCAGCTGCAACTTATTCGA
	CqTTG1-like2	Forward	AAAGGATCCATGGAGAATTCAACCCAAGAATCTCACCTC
		Reverse	AAAGGTCACCTCAAACTCTCAGCAGCTGCAACTTATTC
	AtTTG1	Forward	AAAGAATTCATGGATAATTCAGCTCCAGATTCGTT
		Reverse	AAAGAGCTCTCAAACTCTAAGGAGCTGCATTTTGT
Complementation	*REBC promoter*	Forward	TTGGGCCCGGCGCGCCGAATTCTGCTAAAATTTAATTCATTCTACACATTTT
		Reverse	CATGCGGCGTTGGAACTAGGTTTTTGGG
	REBC CDS	Forward	CCTAGTTCCAACGCCGCATGCACGCACACACAAAGATTTCC
		Reverse	GGAAATTCGAGCTGGTCACCCTATTTAAGACTACGACATTGAAGC
Protein production	*REBC*	Forward (1st PCR)	GAGGTACTTTTCCAAGGTCCGATGCACGCACACACAAAGATTTCCC
		Forward (2nd PCR)	GGGGGATCCCTTGAGGTACTTTTCCAAGGTCCG
		Reverse	CCCCGTCGACCTATTTAAGACTACGACATTGAA
EMS mutation check	*rebc1 position*	Forward	GCATGGTTTTGCATGCATATATACCCCCTAGCTCC
		Reverse	ATGGTGCAATATTACATGACAAGCTTATTG
		Sequence	TCTGATGGATGTATAAATTTCTGGGTGCAA
	rebc2 position	Forward	GCATGGTTTTGCATGCATATATACCCCCTAGCTCC
		Reverse	ATGGTGCAATATTACATGACAAGCTTATTG
		Sequence	ATGGATCGCCAGCTCAGTCACAAGAAAAGC
RT-PCR			
Expression in quinoa plant			
	REBC	Forward	ATGCACGCACACACAAAGATTTCC
		Reverse	CTATTTAAGACTACGACATTGAAG
	CqHSP20	Forward	TACCAAGCTTTTCTAGCGGCCGGCGAT
		Reverse	TTAACCAGAGACATCAATAGACTTGAGC
	CqNCED3	Forward	GACTCCCGCAGACTCTATTTTCAACGAG
		Reverse	GAAGCTTGACTGTCGCAACCAGCTC
	CqMON1	Forward	AGGACATCTGAGTCTTTCTCACCAATATGC
		Reverse	CAGCAAGGGGGTCAAATGCAGCATATA
Arabidopsis transformants			
	REBC	Forward	ATGCACGCACACACAAAGATTTCCCTAGT
		Reverse	AAGGACATGGCTGTGTTCACCATAAA
	AtTTG1	Forward	ATGGATAATTCAGCTCCAGATTCGTT
		Reverse	TCAAACTCTAAGGAGCTGCATTTTGT
	CqTTG1-like1	Forward	AATTTAACTCAAGAATCTCACCTCCTTCCT
		Reverse	GGATAGGGTTAGGGTTAGGGTTTCTTCA
	CqTTG1-like2	Forward	AATTCAACCCAAGAATCTCACCTCCTTCCA
		Reverse	GAGAGATTTTGAGTTAGGGTTTGTTCG
	AtEF1a	Forward	CAGGCTGATTGTGCTGTTCTTATCAT
		Reverse	GGTGGGTACTCGGAGA
