# Supplementary material for: A novel WD40-repeat protein involved in formation of epidermal bladder cells in the halophyte quinoa
Source: Commun Biol. 2020 Sep 17;3:513. doi: 10.1038/s42003-020-01249-w (PMC7498606; doi:10.1038/s42003-020-01249-w)
Supplement: Supplementary file 2 — Description of Additional Supplementary Items [file 42003_2020_1249_MOESM2_ESM.pdf]

Description of additional supplementary items

Supplementary Data 1: Summary of sequence reads for genes downregulated at least 2-fold in *rebc* mutants.

Supplementary Data 2: Summary of sequence reads for genes upregulated at least 2-fold in *rebc* mutants.

Supplementary Data 3: Summary of genes downregulated at least 2-fold in *rebc* mutants.

Supplementary Data 4: Summary of genes upregulated at least 2-fold in *rebc* mutants.

Supplementary Data 5: Source data for the main figures, Supplementary figures, and Supplementary Table 1.
