# Supplementary material for: A novel WD40-repeat protein involved in formation of epidermal bladder cells in the halophyte quinoa
Source: Commun Biol. 2020 Sep 17;3:513. doi: 10.1038/s42003-020-01249-w (PMC7498606; doi:10.1038/s42003-020-01249-w)
Supplement: Supplementary file 8 — Reporting Summary [file 42003_2020_1249_MOESM8_ESM.pdf]

nature research

Corresponding author(s): Tomohiro Imamura and Masashi Mori

Last updated by author(s): Aug 6, 2020

Reporting Summary

Nature Research wishes to improve the reproducibility of the work that we publish. This form provides structure for consistency and transparency in reporting. For further information on Nature Research policies, see Authors & Referees and the Editorial Policy Checklist .

Statistics

For all statistical analyses, confirm that the following items are present in the figure legend, table legend, main text, or Methods section.

n/a	Confirmed	
☐	☒	The exact sample size (*n*) for each experimental group/condition, given as a discrete number and unit of measurement
☐	☒	A statement on whether measurements were taken from distinct samples or whether the same sample was measured repeatedly
☐	☒	The statistical test(s) used AND whether they are one- or two-sided<br>*Only common tests should be described solely by name; describe more complex techniques in the Methods section.*
☒	☐	A description of all covariates tested
☒	☐	A description of any assumptions or corrections, such as tests of normality and adjustment for multiple comparisons
☐	☒	A full description of the statistical parameters including central tendency (e.g. means) or other basic estimates (e.g. regression coefficient) AND variation (e.g. standard deviation) or associated estimates of uncertainty (e.g. confidence intervals)
☐	☒	For null hypothesis testing, the test statistic (e.g. *F*, *t*, *r*) with confidence intervals, effect sizes, degrees of freedom and *P* value noted<br>*Give P values as exact values whenever suitable.*
☒	☐	For Bayesian analysis, information on the choice of priors and Markov chain Monte Carlo settings
☒	☐	For hierarchical and complex designs, identification of the appropriate level for tests and full reporting of outcomes
☒	☐	Estimates of effect sizes (e.g. Cohen's *d*, Pearson's *r*), indicating how they were calculated

Our web collection on statistics for biologists contains articles on many of the points above.

Software and code

Policy information about availability of computer code

Data collection	Genes sequences, involved in this study, were obtained from QGDB (quinnoa genome data base, http://quinoa.kazusa.or.jp/), Pytozome12 (https://phytozome.jgi.doe.gov/pz/portal.html#!info?alias=Org_Cquinoa_er) and NCBI database (https://www.ncbi.nlm.nih.gov/).
Data analysis	In silico substraction used customized Python scripts (https://github.com/Comai-Lab/kmer-extract-by-trigger-site). Mutmap analysis used MutMap pipeline ver. 1.4.4 (http://genome-e.ibrc.or.jp/home/bioinformatics-team/mutmap). For RNA-Seq analysus, the reads were aligned to the Cq_PI614886_genome_V1_pseudomolecule reference sequence (http://www.cbrc.kaust.edu.sa/chenopodiumdb/) by HISAT2. After the gene was predicted by StringTie50 using alignment data from HISAT2, the expression levels of each gene in each sample were compared using featureCounts. The three-dimensional structure of the REBC protein was predicted using Modeller ver. 9.16 and Phyre2 and displayed using MOLMOL. Subcellular localization of the selected gene products was predicted using WolF PSORT (https://wolfpsort.hgc.jp/). MEGA programme version7.0 software were used for genes phylogenetic tree analysis.

For manuscripts utilizing custom algorithms or software that are central to the research but not yet described in published literature, software must be made available to editors/reviewers. We strongly encourage code deposition in a community repository (e.g. GitHub). See the Nature Research guidelines for submitting code & software for further information.

Data

Policy information about availability of data

All manuscripts must include a data availability statement. This statement should provide the following information, where applicable:

- Accession codes, unique identifiers, or web links for publicly available datasets
- A list of figures that have associated raw data
- A description of any restrictions on data availability

All data generated or analyzed during this study are included in this published article (and its supplementary information files).

Field-specific reporting

Please select the one below that is the best fit for your research. If you are not sure, read the appropriate sections before making your selection.

- [x] Life sciences
- [ ] Behavioural & social sciences
- [ ] Ecological, evolutionary & environmental sciences

For a reference copy of the document with all sections, see nature.com/documents/nr-reporting-summary-flat.pdf

Life sciences study design

All studies must disclose on these points even when the disclosure is negative.

Sample size	For the transgenic analysis using Arabidopsis, 10 or more independent T1 plants were produced in each line.
Data exclusions	No data was excluded
Replication	All experimental findings were reliably reproduced.
Randomization	For abiotic-stress treatments, plants of different genotypes were placed randomly.
Blinding	N/A

Reporting for specific materials, systems and methods

We require information from authors about some types of materials, experimental systems and methods used in many studies. Here, indicate whether each material, system or method listed is relevant to your study. If you are not sure if a list item applies to your research, read the appropriate section before selecting a response.

Materials & experimental systems

n/a	Involved in the study	
☐	☒	Antibodies
☒	☐	Eukaryotic cell lines
☒	☐	Palaeontology
☒	☐	Animals and other organisms
☒	☐	Human research participants
☒	☐	Clinical data

Methods

n/a	Involved in the study	
☒	☐	ChIP-seq
☒	☐	Flow cytometry
☒	☐	MRI-based neuroimaging

Antibodies

Antibodies used	anti-SNGDDDTEDTNSAG: produced in rabbit from synthesized SNGDDDTEDTNSAG peptide.
Validation	Validated by western blots on purified REBC protein form Escherichia coli and plant cells.
